# GP consultations for respiratory tract infections in children aged <5 years: a retrospective cohort study 2016–2023

**DOI:** 10.3399/BJGP.2024.0501

**Published:** 2025-06-17

**Authors:** Kimberley Foley, Dougal Hargreaves, Alex Bottle, Jennifer K Quint, Azeem Majeed, Sejal Saglani, Sonia Saxena

**Affiliations:** Department of Primary Care and Public Health, Imperial College London, London; Mohn Centre for Children’s Health and Wellbeing, Imperial College London, London; Centre for Paediatrics & Child Health, Imperial College London, London; Department of Primary Care and Public Health, Imperial College London, London; Department of Primary Care and Public Health, Imperial College London, London; Department of Primary Care and Public Health, Imperial College London, London; Centre for Paediatrics & Child Health, Imperial College London, London; National Heart and Lung Institute, Imperial College London, London; Department of Primary Care and Public Health, Imperial College London, London

**Keywords:** child, COVID-19, primary health care, general practice, respiratory tract infections, *Streptococcus* group A

## Abstract

**Background:**

Little is known about how GP consultation rates for children’s respiratory tract infections (RTIs) have changed since the COVID-19 pandemic restrictions lifted.

**Aim:**

To describe changes in GP consultation rates for RTIs in children aged <5 years from 2016 to 2023.

**Design and setting:**

A population-based retrospective cohort study using electronic health records from primary care practices across England.

**Method:**

All children aged <5 years registered with a general practice in the Clinical Practice Research Datalink Aurum database from April 2016 to March 2023 were included. Monthly GP consultation rates for RTIs from April 2021 to March 2023 were compared with the corresponding months during pre-pandemic years (April 2016 to February 2020).

**Results:**

There were 3 226 285 GP consultations for RTIs among 2 894 539 children. Pre-pandemic, mean monthly consultation rates ranged from lows in August to highs in November (from 2368 to 8682 per 100 000 children, respectively). Following the pandemic lockdowns in 2020, monthly rates in 2021/2022 peaked in June and October at 5152 and 5942 per 100 000 children, respectively, but the winter peak was less marked than pre-pandemic and mean monthly rates were 16.8% lower (95% confidence interval [CI] = − 13.4 to − 19.6). In 2022/2023, after all restrictions were lifted, rates remained around 15% below pre-pandemic years, but the winter peak for children aged 3–4 years was 8615 per 100 000 children, exceeding mean pre-pandemic winter peaks of 6011 per 100 000 children. This was an increase of 43.3% and coincided with a streptococcal group A outbreak. Across all ages there was a sharp increase (from 1486 to 2370 per 100 000 children, around 60%) in tonsillitis, *Streptococcus* A, and bacterial ear infections.

**Conclusion:**

This study shows reductions in GP consultations for RTIs in children aged <5 years since the lifting of COVID-19 pandemic restrictions. Of concern is a sharp rise in tonsillitis, *Streptococcus* A, and bacterial ear infections that should be monitored.

## Introduction

Respiratory tract infections (RTIs) are the most common reason for children aged <5 years to have contact with primary care in the UK and account for approximately one-third of all consultations with children.^1^ Most RTIs affect the upper respiratory tract and cause mild, self-limiting illness. A small proportion affect the lower respiratory tract causing local or systemic infection. Immunisation programmes against respiratory pathogens have reduced the incidence of serious bacterial infections.^2^

In the UK’s publicly funded NHS, approximately 98% of children are registered with an NHS GP who provide first contact care for common childhood illness, with visit rates of 3–6 times per year.^3^ Hence, the NHS model of accessible primary care free at the point of use means that few children need private health care, which usually excludes cover for primary care consultations.^4^,^5^ Therefore, when a child is unwell, parents/carers are encouraged to seek primary care in the first instance. The emergence of the COVID-19 pandemic in 2020 and subsequent national lockdowns and other social restrictions to reduce transmission of the SARS-CoV-2 virus reduced transmission of all respiratory viruses.^6^

During spring of 2020, in compliance with social restrictions, GP practices restructured services to facilitate access.^7^ To manage demand, children and families were encouraged to manage minor illnesses at home. These changes contributed to reduced healthcare activity in 2020/2021 that has been reported extensively.^8^–^12^ However, less is known about the impact of the pandemic on GP consultations for RTIs after the series of lockdowns ended in March 2021.^13^ Encouraging self-management of minor illness at home accompanied by wider availability of resources outside of primary care may have led to sustained changes in health-seeking behaviour.^14^

Reducing transmission of pathogens during the pandemic restrictions lowered the number of reported infections as intended but may have also altered children’s development of naturally acquired immune responses to common respiratory pathogens of childhood. For example, reduced antibody levels to respiratory syncytial virus (RSV) have been reported.^15^

**How this fits in**

GP consultations for respiratory tract infections in children aged <5 years were around 15% below pre-pandemic rates after the lifting of the COVID-19 pandemic restrictions in England. However, by December 2022, the winter peak exceeded pre-pandemic rates with a 43.3% increase (from 6011 to 8615 per 100 000 children) in children aged 3–4 years driven by increased consultations for tonsillitis, *Streptococcus* A, and bacterial ear infections. Children’s respiratory tract infections warrant careful monitoring and, if these patterns persist, GPs could consider increased likelihood of bacterial infections in children presenting with respiratory symptoms in forthcoming winter seasons.

As a result of universal coverage, it is possible to use longitudinal health records to examine health service use such as GP consultation rates. This provides information on changes in prevalence of disease and on health-seeking behaviour. Furthermore, by comparing consultation rates for transmissible and non-transmissible infections, insights can be gained about whether differences are related to incidence/prevalence of disease or health-seeking behaviour. The aim of this study was to describe changes in GP consultation rates for RTIs in children aged <5 years in England from 2016 to 2023.

## Method

### Design and study population

A retrospective cohort study was conducted using electronic health records from the Clinical Practice Research Datalink (CPRD) Aurum database. CPRD Aurum contains electronic health record data collected from GP practices in England, covering over 20% of the population. It is generally representative of the population in terms of age, gender, deprivation status, and geographical distribution.^16^

An open cohort of all children aged <5 years registered with a participating GP practice from 1 April 2016 to 31 March 2023 was created. Children who were registered at the start of the study period were included, adding those joining from the date of their registration with a GP practice. Children were only included if they had an ‘acceptable’ flag recorded in the database indicating that their data met quality standards for key variables.^16^ All children were followed up until they reached the age of 5 years, de-registered from the GP practice, died, or reached the end of the study period, whichever came first.

### Exposure

The pre-pandemic period was defined in this study as 1 April 2016 to 29 February 2020. March 2020 was excluded as things changed rapidly during this month, and it covered both the pre-pandemic and the pandemic period. The time period after the initial pandemic lockdowns from 1 April 2021 to 31 March 2023 was examined, with the first and second fiscal years examined separately.

### Outcomes

The primary outcome was monthly GP consultation rates for all RTIs in children aged <5 years. The study focused on all RTIs as these account for the majority of GP consultations with children and are very common.^17^ The code list was developed to include common RTIs for children including bronchiolitis, viral, and bacterial infections using the CPRD Aurum code browser and existing lists from prior publications and code list repositories (see Supplementary Table S1 for code lists).^3^,^18^–^23^ Consultations included both face-to-face and remote contacts and were defined using administrative and clinical codes following an algorithm previously developed by the authors of the current study.^12^ GP consultations were identified using codes for staff identity and for the primary analysis consultations with allied health professionals (AHPs) were excluded. The first consultation with a GP was included if there were multiple ones recorded on the same day because there was a large increase in remote consultations during the pandemic.^12^ Secondary outcomes included selected bacterial infections (community-acquired pneumonia, bacterial otitis media, and tonsillitis). Infections associated with invasive group A *Streptococcus* disease were also included because of changes in the incidence during the study period.^24^,^25^ For comparison, rates of urinary tract infections (UTIs) were examined.

### Data analysis

Monthly rates were calculated by summing the number of consultations in each month divided by the total registered population in April of each calendar year.

The pre-pandemic trends were plotted and then the mean monthly rate calculated for the pre-pandemic period from April 2016 to February 2020, which was then used as the expected rate. The mean monthly rate that was the highest was used as the pre-pandemic winter peak. The percentage change was calculated by comparing the observed and expected monthly rates from April 2021 to March 2023.

Outcomes were reported for subgroups of age (0 to <1, 1 to <2, 2 to <3, 3 to <4, and 4 to <5 years) and by type of infection.

As the use of AHPs in primary care is increasing,^26^ a sensitivity analysis was undertaken to examine consultation rates for children’s RTIs including both GPs and AHPs. AHPs included nurse practitioners, healthcare assistants, paramedics, pharmacists, midwives, health visitors, and other practitioners.

## Results

There were 2 894 539 children aged <5 years registered with a GP practice during the study period. From fiscal years 2016/2017 to 2022/2023 the number of registered children steadily declined by around 6% from 911 348 in 2016/2017 to 855 542 in 2022/2023 (see Supplementary Table S2). There were 3 226 285 consultations for RTIs.

**Figure 1:**
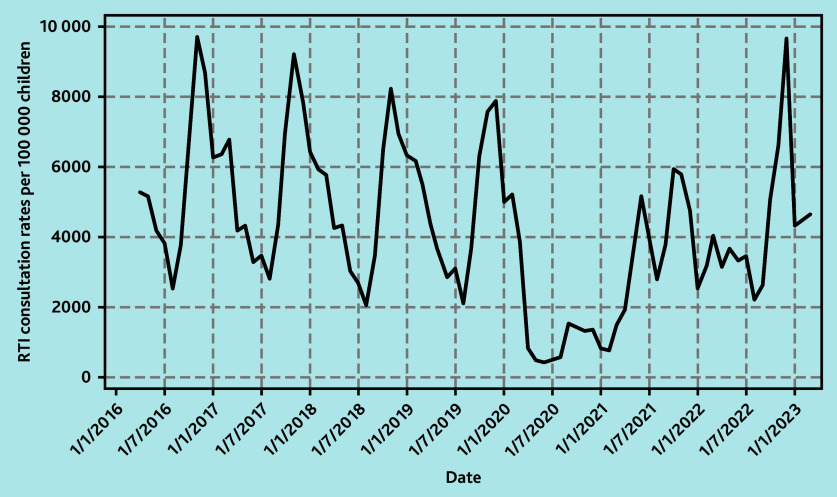
Monthly GP consultation rates for RTIs per 100 000 children aged <5 years. RTI = respiratory tract infection.

In the pre-pandemic period from 1 April 2016 to 29 February 2020 there was a regular pattern of winter peaks in GP consultations for RTIs in children aged <5 years. Mean monthly rates ranged from lows of 2368 per 100 000 children in August to highs of 8682 per 100 000 children in November. There was a gradual decline in the winter peaks pre-pandemic of around 6.5% per year from a winter peak of 9715 per 100 000 children in 2016 to a peak of 7876 per 100 000 children in 2019. This pattern was disrupted during the COVID-19 pandemic in 2020/2021 when mean monthly consultation rates for RTIs in children aged <5 years dropped by about 80% to a low of 423 per 100 000 children in June and a high of 1521 per 100 000 children in September (Figure 1).

In 2021/2022, mean monthly GP consultation rates for RTIs in children aged <5 years were 16.8% (95% confidence interval [CI] = −13.4 to −19.6) lower than expected when compared with mean monthly rates in pre-pandemic years. The lowest monthly rate was in April at 1924 per 100 000 children but comparable with pre-pandemic mean lows of 2368 per 100 000 children that occurred in August. There was a bimodal pattern in June and October with peaks at 5152 and 5942 per 100 000 children, respectively, although these were lower than the pre-pandemic winter peaks. In 2022/2023, mean monthly consultation rates remained 14.9% (95% CI = −11.8 to −17.5) lower than pre-pandemic years although ranging from a low of 2190 per 100 000 children in August to a high of 9644 per 100 000 children in December. From 2016 to 2020, the mean winter peak (in children’s RTI consultations) occurred in November. However, in 2022, the winter peak occurred in December at 9644 per 100 000 children compared with a pre-pandemic mean winter peak of 8682 per 100 000 children. This exceeded pre-pandemic rates by 11.1% (95% CI = 1.90 to 22.1) (Figure 1).

**Figure 2:**
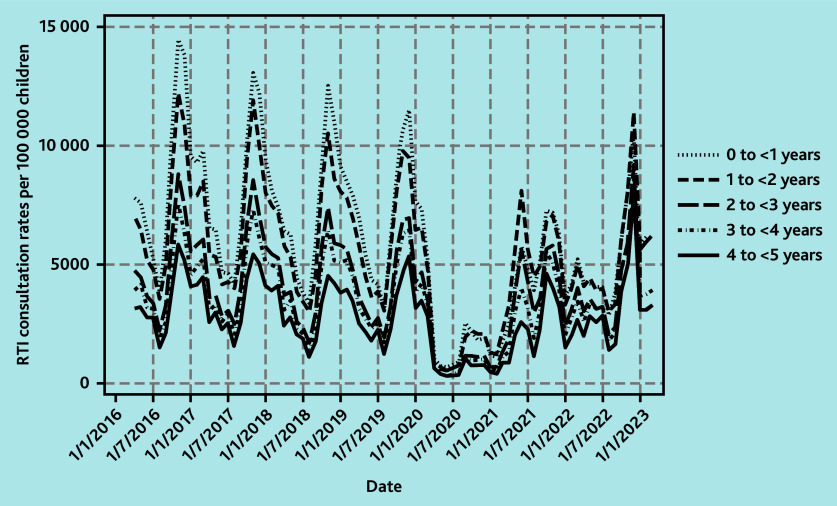
Monthly GP consultation rates for RTIs in children, by age. RTI = respiratory tract infection.

**Figure 3:**
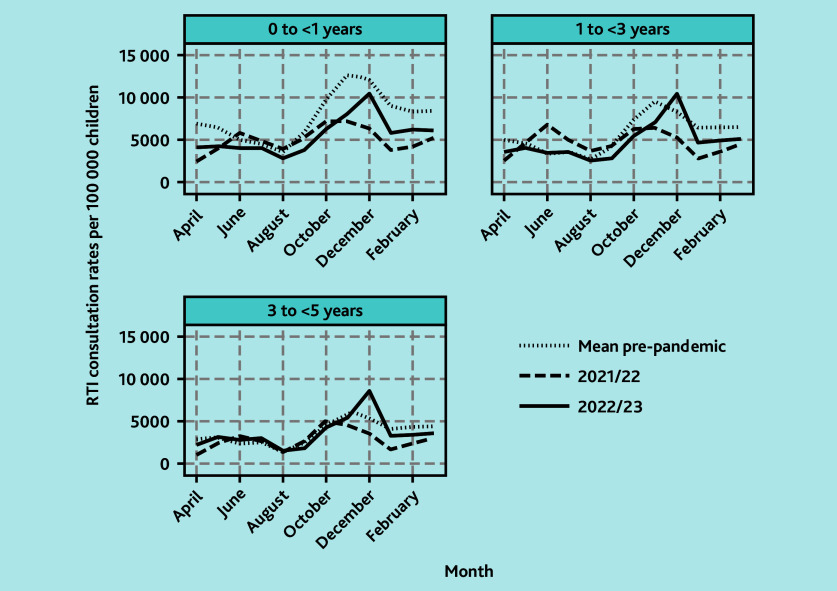
Trends in monthly GP consultation rates for RTIs in the pre-pandemic and post-pandemic lockdown periods (by age group and fiscal year, April to March). RTI = respiratory tract infection.

For children aged <1 year, this age group had the highest mean monthly consultation rates for RTIs pre-pandemic (Figure 2). In 2021/2022, mean monthly consultation rates were 28.3% (95% CI = −25.4 to −30.7) lower overall with an October/November peak of 7247 per 100 000 children and a second, but slightly lesser, June peak of 5771 per 100 000 children. In 2022/2023, there was a winter peak in December of 10 442 per 100 000 children although this was 17.8% (95% CI = −7.27 to −25.4) lower than the mean pre-pandemic winter peak for infants at 12 699 per 100 000 children (Figure 3).

Patterns among children aged 1 to <2 and 2 to <3 years were similar to each other (Figure 2) and these ages are described together. In 2021/2022, mean monthly consultation rates for RTIs were 7.2% (95% CI = −3.13 to −10.5) lower than pre-pandemic, with peaks in November and June of 6424 and 6866 per 100 000 children, respectively, although the June peak was slightly higher (Figure 3). In 2022/2023, although late spring/summer rates were similar to pre-pandemic, mean monthly rates were 14.9% (95% CI = −11.7 to −17.3) lower overall. In December 2022 there was a winter peak of 10 373 consultations per 100 000 children, exceeding the mean pre-pandemic winter peak of 9528 per 100 000 children by 8.9% (95% CI = −0.31 to 19.9).

Patterns among children aged 3 to <4 and 4 to <5 years were similar (Figure 2) and are described together. In 2021/2022, rates were 17.8% (95% CI = −13.9 to −20.7) lower overall and, similar to other ages, there was a bimodal pattern with peak in June/October of 3288 and 5091 per 100 000 children, respectively. However, for these children aged 3 to <5 years, the peak was much higher in October. In 2022/2023, mean monthly rates were similar to pre-pandemic at only 1.4% (95% CI = −4.5 to 2.6) lower, but there was a large winter peak in December 2022 of 8615 per 100 000 children, which was an increase of 43.3% (95% CI = 31.6 to 57.3) compared with mean pre-pandemic winter peaks of 6011 per 100 000 children (Figure 3).

**Figure 4:**
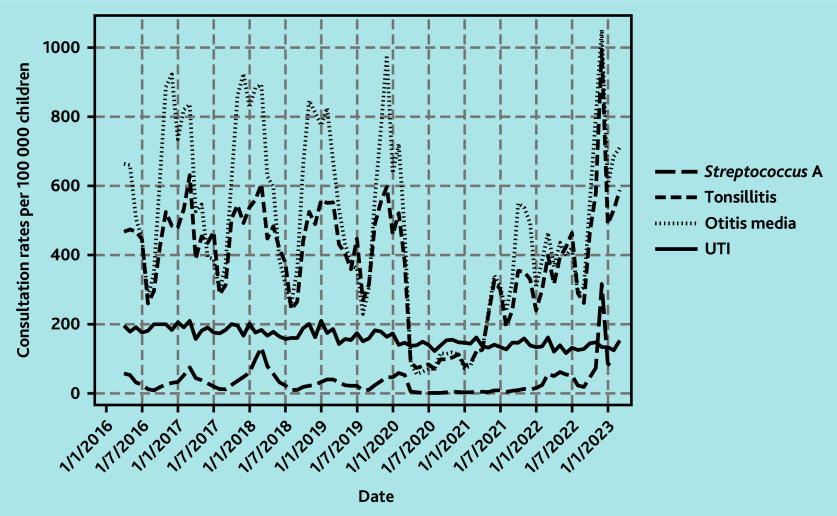
Monthly GP consultations rates for selected upper bacterial infections compared with UTIs. UTI = urinary tract infection.

**Figure 5:**
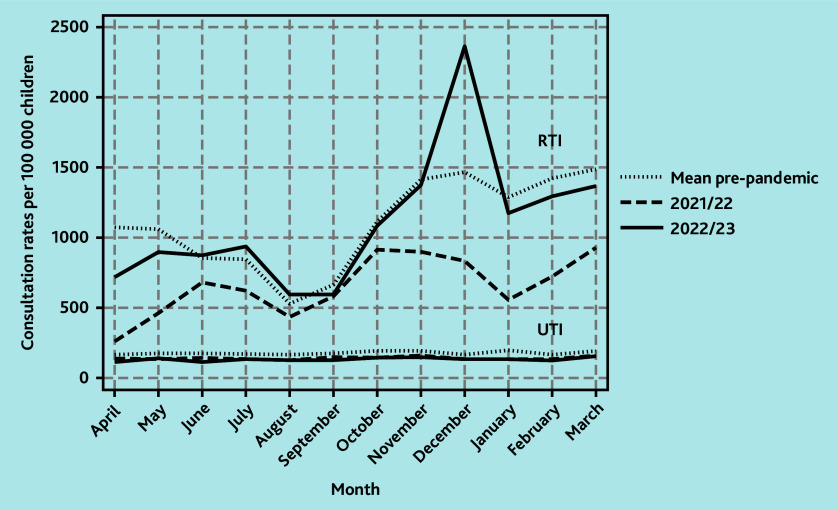
Trends in monthly consultation rates for selected upper bacterial RTIs and UTIs in the pre-pandemic and post-pandemic lockdown periods. RTI = respiratory tract infection. UTI = urinary tract infection.

Trends among specific bacterial infections (bacterial otitis media, tonsillitis, and *Streptococcus* A) for all children aged <5 years are shown in Figure 4. Community-acquired pneumonia was examined separately from the upper respiratory bacterial infections and the overall rates for pneumonia were very low, therefore the focus here is on describing trends in upper bacterial infections. There were major changes in the pattern of GP consultations, with a large peak in December 2022 of 2370 per 100 000 children, which exceeded mean pre-pandemic winter peaks of 1486 per 100 000 children by 59.7% (95% CI = 45.2 to 86.0) (Figure 5). When stratified by the specific type of RTI, this increase was greatest among consultations for *Streptococcus* A at a rate of 317 per 100 000 children in December 2022 compared with a pre-pandemic peak rate of 83 per 100 000 children in March, which is an increase of 3.8 times (Figure 4).

Contrary to these patterns, GP consultation rates for UTIs in children aged <5 years steadily declined by an average of 0.51% (95% CI = −0.54 to 0.49) per month over the study period (Figure 4) and trends in monthly GP consultation rates were similar across time periods, although rates in 2021/2022 and 2022/2023 were lower overall (Figure 5).

Across the study period, 85% (*n* = 3 226 285/3 797 078) of all RTI consultations in children aged <5 years were with a GP. However, the proportion of children’s RTI consultations with AHPs increased from 16% (*n* = 19 310/121 013) to 19% (*n* = 105 928/561 351) between 2020/2021 to 2022/2023. Children’s RTI consultations including both GPs and AHPs showed similar patterns of reductions compared with pre-pandemic consulting rates (see Supplementary Figure S1).

## Discussion

### Summary

The current study shows 15%–17% reductions in GP consultations for RTIs for children aged <5 years from April 2021 to March 2023 compared with pre-pandemic years. By 2022/2023, although mean monthly rates remained approximately 15% lower overall compared with pre-pandemic years, there was a sharp rise (around 60%) in tonsillitis, *Streptococcus* A, and bacterial ear infections.

### Strengths and limitations

The strengths of the current study include its large size and inclusion of a population that is broadly representative of children in England.^16^ This allowed comparison across time periods including before and after the start of the COVID-19 pandemic. The results are therefore highly unlikely to be because of chance or sample selection bias. There are, however, some limitations of the study. As is common with large observational studies using electronic health records, there can be concerns with data accuracy and completeness. For example, GPs may be coding consultations as bacterial instead of viral RTIs to justify antibiotic prescribing.^27^ In this study, this would be less likely to affect overall patterns of RTIs, but may affect the results when reporting by specific RTIs. UTIs were included as a comparator to gain insights about whether differences in children’s RTI consultation rates were because of disease circulation or health-seeking behaviour, but this assumes that health-seeking behaviours are similar across illnesses.

### Comparison with existing literature

The current findings of 15%–17% reductions in GP consultations for children after lockdown restrictions ended are consistent with reports of reduced consultations in children aged 0–4 years in 2021/2022.^28^ They may be explained in part by changes in RTI incidence and immunity that have been reported elsewhere.^29^–^34^ The reduced winter peak for infants in 2022/2023 compared with pre-pandemic years is consistent with initial reports on healthcare activity in 2022/2023 of lower admissions to hospital for RSV^35^ and reduced GP consultations for chickenpox.^36^ Ongoing monitoring of RTIs, including RSV, is essential^37^ as children may now be contracting illnesses at an older age, although infections in infants are likely to be reduced with the introduction of a new maternal vaccine.^38^ Delayed exposure to viral illness during childhood, when immunity is more developed, may be beneficial.^39^,^40^ However, some viruses such as chickenpox result in more severe infections at older ages.^36^ Any change in the exposure to respiratory pathogens in the early years can have a large impact on future lung health^41^,^42^ and risk of premature death from respiratory disease,^43^,^44^ and therefore needs monitoring.

The current findings of sharp increases in GP consultations for *Streptococcus* A infections and RTIs in December 2022 is consistent with both surveillance data^45^ and reports from hospital admissions in the UK.^46^ The pandemic interrupted the pattern of *Streptococcus* A peaks that occur in 3–4 yearly cycles, with the last outbreak in 2018 as observed in the current data (Figure 4) and reported in surveillance data.^47^ However, it is important to consider the impact of changes in clinical guidance related to *Streptococcus* A infections^48^ and subsequent public health messaging urging parents/carers to contact healthcare providers if their child was unwell,^49^ as this may have resulted in an increase in consultations during this outbreak.

Reductions in GP consultations for RTIs may also indicate there have been changes in health-seeking behaviour. During the pandemic, amid signposting towards self-management of viral illness and messaging for the public to stay at home and conserve NHS resources except for serious illness, there was sometimes a misconception that GPs were not available for consultations.^50^ Parents/carers may now have a different perception about the need to consult a GP for RTIs and/or parents may be accessing a wider availability of resources, such as pharmacies and the internet.^36^

Of note, the observed reductions in children’s RTI consultations after lockdown restrictions ended occurred for GPs and AHPs alike. Hence, these falls were not explained by shifting professional roles within the workforce. Moreover, parents of young children who may not have experienced typical RTIs during the pandemic may have been more inclined to seek care when their children became ill,^37^ but this is not supported by the current findings in terms of increasing RTI consultations for infants compared with pre-pandemic. Parents/carers may have also changed how they used the healthcare system in recent years, turning to medical helplines such as NHS 111 or urgent and emergency care outpatient services instead of GP surgeries as the first point of contact. The study found rates of UTIs were slightly lower in 2021–2023 than pre-pandemic, which could suggest general changes in health-seeking behaviour resulting in presentation to urgent and emergency care from delaying access to care.^51^ A shift in care towards the private sector during the pandemic is possible, but this is unlikely. Often care from private healthcare services is used when there are perceived or actual waiting lists for care, but most RTIs are self-limiting and resolve quickly. Furthermore, a study examining the use of private care during the pandemic found that healthcare use decreased.^52^

### Implications for research and practice

The reported falls in children’s RTI consultations may be because of falls in incidence. However, the winter peaks and sharp rises in GP consultations for bacterial ear and throat infections observed in late 2022 suggest GPs could consider the likelihood of bacterial infections when triaging, assessing, and managing children presenting with RTIs. The findings have important implications for NHS general practice in areas such as workforce planning, public health interventions, digital health integration, health equity, and future research priorities.

If the falls in children’s RTI consultations after the pandemic lockdowns ended continue and mean that parents and carers are now managing RTIs without consulting a GP, then they need to know when and how to seek health care when their child is unwell.^53^–^55^ Childhood vaccination rates, which have declined in recent years, need to be maintained, and it is important to target and tailor messages to reach all groups to avoid widening disparities.^56^–^58^

If the alternative explanation for falls in children’s RTI consultations are issues with access to primary care, then system-wide improvements in access are needed. GP numbers have not kept up with population growth, resulting in an increase in the average number of patients per GP. Demand for appointments has also increased in the past 5 years with falling GP numbers.^59^ Previous research has reported that pressures on GP appointments mean that those with lower access to GPs have more children’s visits to emergency departments.^60^ Additional support for the primary care workforce is essential.^61^

This study found 15%–17% reductions in GP consultations for RTIs after lockdown restrictions ended compared with pre-pandemic. This may be explained by a combination of changes in incidence of RTIs, health-seeking behaviour, and/or patterns of health service use.^62^ Capacity constraints in NHS general practice and difficulty in making appointments may also play a role. The long-term impact of reduced exposure to typical RTIs for young children is not known, but by using the pandemic as a natural experiment, it is possible to examine critical time windows in immune system development^43^,^63^ to better understand how later exposure to RTIs may affect the immune system development and development of lung health.

The authors of this study recommend future research to examine how reductions in GP consultations for children’s RTIs has an impact on pre-existing health inequalities, particularly among families with poor access to services or low health literacy, and to explore whether antibiotic prescribing for RTIs and referrals to secondary care mirror consultation patterns in this age group. These patterns of children’s RTI consultations warrant careful monitoring to observe a clearer trajectory that will assist in planning for health service pressures in peak periods. Future research could also investigate opportunities to incorporate predictive analytics and digital tools, such as symptom triage apps and teleconsultation platforms, into primary care pathways. These could support early identification of severe infections, improve patient flow, and reduce unnecessary in-person consultations, particularly during peak periods when demand for GP appointments is high.

Some general practices actively audit and plan for appointment availability to cope with winter pressures. Practices should consider seasonal audits of appointment availability and flexible staffing models to cope with winter pressures. Expanding the roles of AHPs in managing RTIs could alleviate GP workloads, provided appropriate training and support are in place.

Having strong primary care is important to address current challenges and future shocks to the health system. Specific areas that require strengthening include the primary care workforce,^61^ digital systems that support responsive research and integration across primary and secondary care,^64^ and making sure access to primary care is maintained for all population groups, including children. Policymakers should prioritise establishing models of care delivery that account for population growth and seasonal variations in demand. Strengthening the primary care workforce and integrating care pathways between primary and specialist services are critical to ensuring equitable access for all.

## Supplementary Information



## Data Availability

Additional unpublished data related to this study are not available. Access to CPRD data is only available to researchers with approved protocols via CPRD’s Research Data Governance Process. Code lists are available in Supplementary Table S1 and are shared without investigator support.
