# Supplementary material for: GP consultations for respiratory tract infections in children aged <5 years: a retrospective cohort study 2016–2023
Source: Br J Gen Pract. 2025 Jun 17:BJGP.2024.0501. Online ahead of print. doi: 10.3399/BJGP.2024.0501 (PMC12199992; doi:10.3399/BJGP.2024.0501)
Supplement: Supplementary file 1 [file BJGP.2024.0501_suppl.pdf]

Supplementary Table S1. Code lists

	MedCodeId	Term
**All respiratory tract infections**	2744861000000110	Suspected Legionella pneumonia
	2724991000000113	Suspected whooping cough
	11823221000006114	Non-productive cough
	11905001000006111	Obliterating fibrous bronchiolitisme
	12717061000006114	Acute bronchitis and/or bronchiolitis
	12728461000006112	Pneumonia due to unspecified organism
	12728631000006117	Bronchopneumonia due to unspecified organism
	13031601000006113	Croup
	4943451000006119	Able to cough up sputum
	2834841000006118	Acute bacterial epiglottitis
	2976201000006115	Acute epiglottitis and supraglottitis
	3805511000006115	Acute bronchitis with bronchospasm
	4353381000006111	Acute bronchopneumonia
	4780481000006116	Acute parainfluenza virus bronchitis
	4780511000006112	Acute bronchitis caused by rhinovirus
	4780571000006115	Acute bronchiolitis caused by respiratory syncytial virus
	5053381000006118	Acute bronchiolitis due to adenovirus
	5053391000006115	Acute bronchiolitis caused by adenovirus
	5055921000006115	Acute toxic tracheobronchitis
	5573791000006115	Acute fibrinous laryngotracheobronchitis
	6015831000006117	Acute infective tracheobronchitis
	7715861000006113	Acute bronchitis co-occurrent with bronchiectasis
	3658911000006115	Adenoviral laryngotracheobronchitis
	5036301000006119	Adult acute epiglottitis and supraglottitis
	7618751000006116	Aspergillus tracheobronchitis
	6566941000006114	Bilateral bronchopneumonia
	3193601000006111	Bout of coughing
	2828791000006112	Brassy cough
	9309111000006113	Bronchopneumonia due to bacteria
	9309121000006117	Bronchopneumonia caused by bacteria
	9309161000006111	Bronchopneumonia due to Group A Streptococcus
	9309171000006116	Bronchopneumonia caused by Group A Streptococcus
	9309181000006118	Bronchopneumonia due to Group B Streptococcus
	9309191000006115	Bronchopneumonia caused by Group B Streptococcus
	9309211000006119	Bronchopneumonia due to Haemophilus influenzae
	9309221000006110	Bronchopneumonia caused by Haemophilus influenzae

9309231000006113	Bronchopneumonia due to Human metapneumovirus
9309241000006115	Bronchopneumonia caused by Human metapneumovirus
9309261000006116	Bronchopneumonia due to Klebsiella pneumoniae
9309271000006111	Bronchopneumonia caused by Klebsiella pneumoniae
9309281000006114	Bronchopneumonia due to methicillin resistant Staphylococcus aureus
9309291000006112	Bronchopneumonia caused by methicillin resistant Staphylococcus aureus
9309301000006113	Bronchopneumonia due to methicillin susceptible Staphylococcus aureus
9309311000006111	Bronchopneumonia caused by methicillin susceptible Staphylococcus aureus
9309331000006117	Bronchopneumonia due to Mycoplasma pneumoniae
9309341000006110	Bronchopneumonia caused by Mycoplasma pneumoniae
9309381000006116	Bronchopneumonia due to Pseudomonas
9309391000006118	Bronchopneumonia caused by Pseudomonas
9309401000006116	Bronchopneumonia due to respiratory syncytial virus
9309411000006118	Bronchopneumonia caused by respiratory syncytial virus
9309421000006114	Bronchopneumonia due to Staphylococcus
9309431000006112	Bronchopneumonia caused by Staphylococcus
9309451000006117	Bronchopneumonia due to Staphylococcus aureus
9309461000006115	Bronchopneumonia caused by Staphylococcus aureus
9309471000006110	Bronchopneumonia due to Streptococcus
9309481000006113	Bronchopneumonia caused by Streptococcus
9309501000006115	Bronchopneumonia due to Streptococcus pneumoniae
9309511000006117	Bronchopneumonia caused by Streptococcus pneumoniae
9309521000006113	Bronchopneumonia due to virus
2737201000006113	Catarrhal laryngitis
8041811000006113	Chronic pharyngolaryngitis
2613771000006117	Cough at rest
5468241000006113	Coughing
5533011000006112	Complaining of cough
6344071000006117	Cough enhancement
6357671000006115	Coughing and deep breathing
3374621000006117	Croupous bronchitis
3657821000006118	Croup syndrome

5259301000006118	Croupy breathing
5754161000006114	Croupy cough
3353911000006119	Decreased coughing
5741451000006116	Does cough
5890991000006118	Does cough up sputum
3518151000006117	Early morning cough
3911121000006113	Edematous laryngitis
5890901000006113	Effective cough
3807341000006118	Epiglottitis
8260311000006113	Episodic dry cough
3193611000006114	Fit of coughing
3014181000006119	Haemorrhagic varicella pneumonitis
3161161000006116	Haemophilus influenzae laryngitis
3451431000006111	Haemophilus influenzae epiglottitis
3474321000006118	Hacking cough
3693751000006118	Haemophilus influenzae laryngotracheobronchitis
3014191000006116	Hemorrhagic varicella pneumonitis
6748141000006119	Human parainfluenza viruses
2784161000006115	Hypertrophic laryngitis
3164871000006118	Influenzal bronchopneumonia
3520051000006112	Increasing frequency of cough
7618761000006119	Invasive Aspergillus tracheobronchitis
5887701000006110	Left basal pneumonia
2961941000006112	Loose cough
5887741000006112	Lobar pneumonia left lower lobe
5887801000006119	Lobar pneumonia right lower lobe
5887841000006117	Lobar pneumonia left upper lobe
5887881000006111	Lobar pneumonia right middle lobe
3316401000006114	LRTI - Lower respiratory tract infection
3896411000006114	LTB - Laryngotracheobronchitis
2961951000006114	Moist cough
3259121000006116	Mycoplasma pneumoniae pneumonia
4063811000006117	Mycoplasmal tracheobronchitis
5053561000006116	Mycobacterial pneumonia
3516981000006112	Nocturnal cough
6781071000006112	Non-productive cough
3304991000006116	Observation of cough
3911111000006117	Oedematous laryngitis
2940361000006114	Parainfluenza virus bronchitis
3069901000006113	Parainfluenza virus laryngotracheobronchitis
3193591000006115	Paroxysmal cough
3193621000006118	Paroxysm of coughing
3555361000006118	Parainfluenza virus bronchopneumonia

3555371000006113	Parainfluenza bronchopneumonia
3555871000006115	Parainfluenza virus pneumonia
3783471000006113	Parainfluenza virus pharyngitis
3895141000006112	Parainfluenza virus laryngitis
5036281000006118	Paediatric acute epiglottitis and supraglottitis
5243651000006110	Painful cough
7069651000006113	Pain provoked by coughing
5036291000006115	Pediatric acute epiglottitis and supraglottitis
7305051000006119	Persistent cough after viral respiratory infection
3482521000006111	Pleurobronchopneumonia
3029801000006112	Pneumococcal laryngitis
3047811000006116	Pneumonia due to Streptococcus
3130351000006119	Pneumonia due to Proteus mirabilis
3153481000006112	Pneumococcal bronchitis
3166801000006115	Pneumonia due to Pseudomonas
3259141000006111	Pneumonia due to Mycoplasma pneumoniae
3259161000006110	Pneumonia caused by Mycoplasma pneumoniae
3334901000006115	Pneumonia due to Escherichia coli
3465971000006110	Pneumonia in whooping cough
3555891000006119	Pneumonia due to parainfluenza virus
5053421000006111	Pneumococcal pneumonia
6782081000006117	Pneumonia due to anaerobic bacteria
6782101000006113	Pneumonia due to aerobic bacteria
6870531000006115	Pneumocystosis jirovecii pneumonia
7119711000006114	Pneumonia due to Gram-negative bacteria
7253251000006114	Pneumonia due to Staphylococcus aureus
7257331000006115	Pneumonia due to infection by Streptococcus pyogenes
7257361000006112	Pneumonia due to infection caused by Streptococcus pyogenes
7618701000006115	Pneumonia due to Schistosoma haematobium
8002221000006116	Pneumonia due to influenza
8038861000006115	Pneumonia due to methicillin resistant Staphylococcus aureus
8040571000006110	Pneumonia due to methicillin susceptible Staphylococcus aureus
8045331000006113	Pneumonia due to H1N1 influenza
8045571000006116	Pneumonia due to avian influenza
4201721000006111	Postural cough
7305041000006116	Postviral cough
3259071000006116	Primary atypical pneumonia due to Mycoplasma pneumoniae
3259171000006115	Primary atypical pneumonia caused by Mycoplasma pneumoniae

3529611000006112	Primary tuberculosis
7487991000006111	Primary tuberculous complex confirmed by culture
7488001000006118	Primary tuberculous complex confirmed histologically
7488011000006115	Primary tuberculous complex confirmed by microscopic examination
7488031000006114	Primary tuberculous complex confirmed
7488041000006116	Primary tuberculous complex with bacteriological or histological examination results unknown
4514601000006116	PTB - Pulmonary tuberculosis
3674671000006113	Respiratory syncytial virus laryngotracheobronchitis
6912171000006113	Respiratory tract congestion and cough
7532981000006114	Respiratory tuberculosis
5887761000006111	Right basal pneumonia
5482021000006114	Sounds clear with cough
3524231000006119	Spasmodic cough
8010731000006112	Spasmodic croup
3845201000006113	Suppurative laryngitis
4514611000006118	TB - Pulmonary tuberculosis
7618741000006118	Tracheobronchitis due to Aspergillus
7618771000006114	Tracheobronchitis caused by Aspergillus
3103051000006117	Ulcerative laryngitis
2688441000006111	Unproductive cough
4943441000006116	Unable to cough up sputum
8045301000006117	Upper respiratory symptoms due to avian influenza
8045311000006119	Upper respiratory symptoms caused by avian influenza
8045361000006116	Upper respiratory symptoms due to H1N1 influenza
8045371000006111	Upper respiratory symptoms caused by H1N1 influenza
8106031000006115	Upper respiratory symptoms due to Influenza A
8106041000006113	Upper respiratory symptoms caused by Influenza A
9316541000006114	Upper respiratory symptoms due to Influenza
9316551000006111	Upper respiratory symptoms caused by Influenza
4781311000006113	Varicella pneumonia
2946761000006112	WC - Whooping cough
3844841000006110	Whooping cough due to organism other than Bordetella pertussis
5526131000006112	Whooping cough-like syndrome
460161011	Influenza-like symptoms
252344014	Respiratory symptoms
82824016	Cough
407081018	C/O - cough
20419011	Dry cough

252351017	Productive cough -clear sputum
252352012	Productive cough -green sputum
252353019	Productive cough-yellow sputum
397882011	Productive cough
598431000006113	Coughing up phlegm
252357018	Night cough present
252359015	Chesty cough
252360013	Bronchial cough
113213012	Chronic cough
423230012	Persistent cough
252363010	Morning cough
252364016	Evening cough
459738011	Unexplained cough
216653013	Cough with fever
103764016	Bovine cough
303321000000115	Reflux cough
30371011	Barking cough
479311018	Cough on exercise
252366019	Cough symptom NOS
252406018	Nocturnal cough / wheeze
105237015	Primary tuberculous infection
146495016	Primary tuberculous complex
878951000006115	Primary tuberculosis infection
286216017	Primary tuberculous infection NOS
239551014	Pulmonary tuberculosis
286221019	Other specified pulmonary tuberculosis
286222014	Pulmonary tuberculosis NOS
286223016	Other respiratory tuberculosis
175131000006112	Respiratory tuberculosis, bacteriologically and histologically confirmed
107761000006118	Tuberculosis of lung, confirmed by sputum microscopy with or without culture
206221000006117	Primary respiratory tuberculosis, confirmed bacteriologically and histologically
168861000006111	Respiratory tuberculosis, not confirmed bacteriologically or histologically
286252018	Other specified respiratory tuberculosis
286257012	Other specified respiratory tuberculosis NOS
46589015	Whooping cough
286448015	Whooping cough - other specified organism
286451010	Other whooping cough NOS
286452015	Whooping cough NOS
141055011	Streptococcal laryngitis

301400016	Varicella pneumonitis
219761000006111	Candidal pneumonia
287630010	Histoplasma duboisii with pneumonia
288040011	[X]Whooping cough, unspecified
961781000006119	Cough
1772711000006118	Basal pneumonia
1772721000006114	Bilateral basal pneumonia
1772771000006110	Bronchopneumonia
1709331000006110	Cough on exercise
1780331000006117	Daytime cough
1772681000006119	Lobar pneumonia
301052010	Acute oedematous laryngitis
301053017	Acute ulcerative laryngitis
301054011	Acute catarrhal laryngitis
301058014	Acute pneumococcal laryngitis
301059018	Acute suppurative laryngitis
456591000006112	Acute epiglottitis (non-streptococcal)
83125011	Acute epiglottitis without obstruction
117891019	Viral epiglottitis
1001621000006118	Croup
301080012	Acute epiglottitis
118208010	Croup
301095014	Acute bronchitis and bronchiolitis
18268014	Acute bronchitis
301100011	Acute fibrinous bronchitis
411490016	Acute wheezy bronchitis
301103013	Acute purulent bronchitis
2475602011	Acute croupous bronchitis
58909014	Acute tracheobronchitis
301105018	Acute pneumococcal bronchitis
301106017	Acute streptococcal bronchitis
301108016	Acute haemophilus influenzae bronchitis
350044014	Acute bronchitis due to mycoplasma pneumoniae
456021000006115	Acute bronchitis due to parainfluenza virus
301116013	Acute bronchitis due to rhinovirus
301119018	Subacute bronchitis
301120012	Acute viral bronchitis
301121011	Acute bacterial bronchitis
301122016	Acute bronchitis NOS
10187013	Acute bronchiolitis
2478755014	Acute capillary bronchiolitis
99508014	Acute obliterating bronchiolitis
25801011	Acute bronchiolitis with bronchospasm

301126018	Acute exudative bronchiolitis
301127010	Obliterating fibrous bronchiolitis
301128017	Acute bronchiolitis due to respiratory syncytial virus
301130015	Acute bronchiolitis NOS
457801000006117	Acute lower respiratory tract infection
885041000006119	Acute bronchitis/bronchiolitis
301132011	Acute bronchitis or bronchiolitis NOS
396090018	Lower respiratory tract infection
546411000006111	Acute lower respiratory tract infection
579878017	Acute lower respiratory tract infection
451444011	Recurrent lower respiratory tract infection
301143015	Chest cold
301241012	Chronic laryngitis and laryngotracheitis
50120012	Chronic laryngitis
301242017	Chronic simple laryngitis
301243010	Chronic catarrhal laryngitis
311731000000118	Congested larynx
555041000006112	Chronic laryngitis NOS
138105010	Chronic laryngotracheitis
301248018	Chronic laryngitis NOS
301357010	Pneumonia and influenza
125510013	Viral pneumonia
301362011	Pneumonia due to respiratory syncytial virus
546491000006118	Chest infection - viral pneumonia
1232627018	Pneumonia due to parainfluenza virus
2872480010	Pneumonia due to Human metapneumovirus
301363018	Viral pneumonia NEC
301364012	Viral pneumonia NOS
546451000006112	Chest infection - pneumococcal pneumonia
885191000006112	Lobar -pneumococcal -pneumonia
301368010	Other bacterial pneumonia
107173015	Pneumonia due to Klebsiella pneumoniae
1222332017	Chest infection - other bacterial pneumonia
219851000006115	Pneumonia due to haemophilus influenzae
219991000006118	Pneumonia due to streptococcus, group B
301370018	Pneumonia due to other specified bacteria
219771000006116	Legionella pneumonia
2765492018	Pneumonia due to Gram negative bacteria
301375011	Pneumonia due to bacteria NOS
301377015	Pneumonia due to other specified organisms
1222333010	Chest infection - pneumonia organism OS
350054013	Chlamydial pneumonia
301382010	Pneumonia due to specified organism NOS

396104016	Pneumonia with infectious diseases EC
220071000006117	Pneumonia with cytomegalic inclusion disease
546511000006112	Chest infection with infectious disease EC
1231963019	Pneumonia with whooping cough
1231962012	Pneumonia with pertussis
885221000006117	Whooping cough pneumonia
1219703018	Pneumonia with aspergillosis
301394018	Pneumonia with other infectious diseases EC
220181000006118	Pneumocystosis pneumonia
301403019	Pneumonia with other infectious diseases EC NOS
301404013	Pneumonia with infectious diseases EC NOS
396105015	Bronchopneumonia
546481000006116	Chest infection - unspecified bronchopneumonia
885231000006119	Bronchopneumonia
301409015	Lobar pneumonia
451130014	Basal pneumonia
546441000006110	Chest infection - pnemonia due to unspecified organism
459416013	Postoperative pneumonia
1229740013	Influenza with bronchopneumonia
546421000006115	Chest infection - influenza with pneumonia
301416019	Influenza with laryngitis
301418018	Influenza with respiratory manifestations NOS
778711000006116	Influenza-like illness
301437010	Tracheobronchitis
142425010	Laryngotracheobronchitis
350041018	Chest infection - unspecified bronchitis
87480013	Chronic tracheobronchitis
52762016	Hypostatic bronchopneumonia
907541000006113	[RFC] Whooping cough
317403015	[D]Cough
2168391000000116	[D]Episodic dry cough
2756071000006111	Viral bronchitis
4200491000006111	Adenoviral bronchitis
3514606010	Protracted bacterial bronchitis
456001000006113	Acute bronchitis due to echovirus
456031000006117	Acute bronchitis due to respiratory syncytial virus
4780541000006111	Acute bronchitis due to echovirus
5153171000006110	Capillaria aerophila chest infection
3524821000006116	Influenza with respiratory manifestation other than pneumonia
5036201000006110	Acute simple laryngitis
2622071000006116	Cytomegaloviral pneumonia

3163841000006112	Adenoviral pneumonia
739941000006111	Pneumococcal lobar pneumonia
5407016	Pneumococcal pleurisy
301376012	Bacterial pneumonia
7705191000006113	Recurrent bacterial pneumonia
3259111000006112	Pneumonia due to PPLO
6870511000006114	PCP - Pneumocystis pneumonia
7823451000006115	Infection of lung caused by Pneumocystis
220251000006118	Chickenpox pneumonia
5887931000006117	Lobar pneumonia right upper lobe
7700581000006111	Postoperative bronchitis
885251000006114	Influenza + pneumonia
1135701000000110	Possible influenza A virus H1N1 subtype
1122831000000118	Suspected Influenza A virus subtype H1N1 infection
301421016	Influenza with other manifestations
301424012	Influenza with other manifestations NOS
301431011	Pneumonia or influenza NOS
396106019	Influenza NOS
301430012	Other specified pneumonia or influenza
778871000006116	Influenza with pneumonia
7267061000006115	Influenza caused by Influenza A virus subtype H1N1
8191011000006119	Possible influenza due to Influenza A virus subtype H1N1
4063911000006112	Influenza like illness
7263131000006117	Influenza A virus subtype H1N1
6207041000006119	Swine influenzavirus
8002231000006118	Pneumonia caused by influenza
6952711000006112	Influenzavirus type A, avian, H1N1 strain
12488541000006113	Influenza with pneumonia, influenza virus identified
6206991000006112	Avian influenzavirus
2609031000000111	Influenza with pneumonia due to seasonal influenza virus
1988661000006110	Influenza with other manifestations due to seasonl inf virus
389891000006119	[X]Influenza+other manifestations, virus not identified
301417011	Influenza with pharyngitis
7792061000006110	Influenza caused by pandemic influenza virus
7264311000006112	Influenza caused by Influenza A virus
12721231000006111	Pneumonia or influenza NOS
7267051000006117	Influenza A (H1N1)
301414016	Influenza with pneumonia NOS
301415015	Influenza with other respiratory manifestation

1707731000006118	Suspected swine influenza
1128141000000118	Influenza A (H1N1) swine flu
6206981000006114	Influenzavirus, type A, avian
3392971000006112	Influenza virus
8045341000006115	Pneumonia caused by H1N1 influenza
7874741000006113	Influenza virus
3555901000006115	Parainfluenza pneumonia
8191021000006110	Possible swine influenza
1707241000006115	Swine influenza
11203012	Influenza
7874751000006110	Influenzavirus
8045581000006118	Pneumonia caused by avian influenza
1988651000006113	Influenza with othr resp manifestation due to seasonal virus
2820795016	Influenza due to Influenza A virus subtype H1N1
3555881000006117	Parainfluenzal pneumonia
9310111000006116	Bronchiolitis caused by influenza virus
301413010	Influenza with pneumonia, influenza virus identified
301423018	Influenza with gastrointestinal tract involvement
4063891000006110	Flu-like symptoms
762181000006116	Flu-like illness
350051017	Atypical pneumonia
3064341000006112	Primary atypical pneumonia
92429016	Avian influenza
6952721000006116	Avian Influenza A H1N1 Virus
1479355018	Community acquired pneumonia
1787121000006116	Community acquired pneumonia
2674072012	Hospital acquired pneumonia
7044731000006115	HAP - hospital acquired pneumonia
1787131000006118	Hospital acquired pneumonia
2163183015	Wheezy bronchitis
183191000006115	Recurrent wheezy bronchitis
301441014	Bronchitis NOS
396107011	Bronchitis
301457014	Other chronic bronchitis
105519017	Chronic bronchitis
11928071000006115	Bronchiolitis obliterans organising pneumonia
11778011000006118	BOOP - Bronchiolitis obliterans organising pneumonia
11778031000006112	BOOP - Bronchiolitis obliterans organizing pneumonia
11777991000006114	Bronchiolitis obliterans organizing pneumonia

4426821000006117	BOOP - Bronchiolitis obliterans organizing pneumonia
4426791000006114	Bronchiolitis obliterans with organizing pneumonia
4426831000006119	BOOP - Bronchiolitis obliterans organising pneumonia
4426801000006110	Bronchiolitis obliterans organizing pneumonia
11778021000006114	Bronchiolitis obliterans with usual interstitial pneumonitis
492443010	Bronchiolitis obliterans
4426811000006113	Bronchiolitis obliterans with organising pneumonia
474828010	Bronchiolitis obliterans organising pneumonia
5054901000006112	Bronchiolitis obliterans with usual interstitial pneumonitis
3524071000006110	Tracheitis
13306011	Chronic tracheitis
301526011	Bronchiectasis NOS
5053681000006113	Idiopathic bronchiectasis
3183591000006116	Bronchiectasis, chronic sinusitis AND dextrocardia syndrome
21163015	Bronchiectasis
301524014	Recurrent bronchiectasis
301525010	Post-infective bronchiectasis
4781641000006119	Postinfectious bronchiectasis
301819015	[X]Other acute lower respiratory infections
555461000006119	Chronic obstructive pulmonary disease with acute lower respiratory infection
8144211000006118	[X]Other acute lower respiratory infections
2807391000006117	Acute empyema
3451101000006114	Empyema thoracis
2990251000006110	Empyema necessitans
3584591000006112	Empyema
301635012	Empyema of pleura
2730071000006112	Tuberculous pleural empyema
5525211000006118	Pleural empyema with fistula
5525231000006112	Empyema of pleura without fistula
218821000006118	Pleural empyema
456436010	Empyema
5525221000006114	Pleural empyema with no fistula
50946012	Empyema with bronchocutaneous fistula
47025013	Empyema with bronchopleural fistula
301647018	Acute dry pleurisy
1231843013	Purulent pleurisy
301644013	Pleurisy
5053821000006113	Dry pleurisy

141663013	Hypostatic pneumonia
2240631000000119	Eosinophilic bronchitis
301809014	[X]Other viral pneumonia
301811017	[X]Other bacterial pneumonia
301812012	[X]Pneumonia due to other specified infectious organisms
301818011	[X]Other pneumonia, organism unspecified
242382011	Aspiration pneumonitis
4514931000006118	Aspiration pneumonitis
301586014	Pneumonitis due to inhalation of food or vomitus NOS
494815013	Pneumonitis due to inhalation of gastric secretions
301579018	Pneumonitis due to inhalation of food or vomitus
3156811000006110	Aspiration pneumonia due to regurgitated gastric secretions
885391000006114	Aspiration pneumonia
3156821000006119	Aspiration pneumonia caused by regurgitated gastric secretions
301585013	Aspiration pneumonia due to vomit
3726611000006119	Aspiration pneumonia caused by regurgitated food
1756121000006110	Aspiration pneumonia
3726601000006117	Aspiration pneumonia due to regurgitated food
7532701000006115	Usual interstitial pneumonia
2475606014	Usual interstitial pneumonitis
301821013	[X]Acute bronchiolitis due to other specified organisms
325070014	Aspiration pneumonia resulting from a procedure
6207041000006119	Swine influenzavirus
1122841000000110	Suspected swine influenza
1707731000006118	Suspected swine influenza
1707241000006115	Swine influenza
301144014	Other specified acute respiratory infections
300997012	Acute respiratory infections
7613991000006116	Severe acute respiratory infection
7614001000006113	Severe acute respiratory infection (SARI)
136463019	Common cold
598191000006116	Acute coryza
681561000006119	Nasal catarrh - acute
3838191000006118	Acute nasal catarrh
195731000006118	Pyrexial cold
162421000006116	Acute rhinitis
3838251000006116	Acute infective rhinitis
486416017	Acute pharyngitis
459581000006119	Acute pharyngitis NOS

301027013	Acute bacterial pharyngitis NOS
301024018	Acute bacterial pharyngitis
301028015	Acute viral pharyngitis
29982014	Acute tonsillitis
301049019	Acute tonsillitis NOS
100101000006117	Throat infection - pharyngitis
100111000006119	Throat infection - tonsillitis
301046014	Acute viral tonsillitis
412576019	Acute laryngitis and tracheitis
412577011	Acute laryngitis/tracheitis
301065018	Acute laryngitis NOS
301082016	Acute laryngitis and/or tracheitis
989171000006116	Acute laryngitis/tracheitis
12028011	Acute laryngitis
15053015	Acute tracheitis with obstruction
301068016	Acute tracheitis NOS
106996011	Acute tracheitis without obstruction
44623012	Acute tracheitis
63301015	Acute laryngotracheitis without obstruction
107009017	Acute laryngotracheitis
99622016	Acute laryngotracheitis with obstruction
301074016	Acute laryngotracheitis NOS
5899261000006111	Acute viral laryngotracheitis
6747021000006112	Infectious laryngotracheitis virus
2671901000006117	Parainfluenza virus laryngotracheitis
91647017	Laryngotracheitis
301083014	Other acute upper respiratory infections
92029013	Acute laryngopharyngitis
362471000006114	[X]Acute upper respiratory infections
3382661000006111	AURTI - Acute upper respiratory tract infection
3203641000006113	Influenzal acute upper respiratory infection
460751000006119	Acute upper respiratory infection
406151000006117	Acute upper respiratory infection of multiple sites
3382671000006116	Acute upper respiratory tract infection
301087010	Pharyngotracheitis
12758201000006114	Pharyngotracheitis
301088017	Recurrent upper respiratory tract infection
301089013	Pharyngolaryngitis
301146011	Other upper respiratory tract diseases
12725541000006119	Other upper respiratory tract diseases NOS
301090016	Other upper respiratory infections of multiple sites
12730221000006118	Other upper respiratory tract diseases
301353014	Other upper respiratory tract diseases NOS

396089010	Upper respiratory infection NOS
3378391000006115	URI - Upper respiratory infection
6013001000006119	Bacterial upper respiratory infection
73091000006118	Upper respiratory infection
5573831000006110	RTI - Respiratory tract infection
5655871000006119	URTI - Viral upper respiratory tract infection
350040017	Viral upper respiratory tract infection
411488017	Respiratory tract infection
3378381000006118	Upper respiratory tract infection
12726371000006114	Acute respiratory infection NOS
175471000006116	Respiratory infection NOS
2578701000006119	Sinus pain
5561911000006117	On examination - maxillary sinus pain
5892621000006114	Maxillary sinus pain
5892631000006112	Frontal sinus pain
5561931000006111	On examination - frontal sinus pain
5561921000006113	O/E -frontal sinus pain
633891000006119	Ear symptom
477607011	Ear pain
918691000006116	'Blocked ear'
4088541000006111	Blocked ears
291801000006117	Sensation of blocked ears
883851000006112	Otalgia - earache
253180011	Bilateral earache
2753831000006111	Earache
253179013	Unilateral earache
633981000006112	Earache symptoms
253182015	Earache symptom
5875371000006118	Ear discharge
5875391000006117	Observation of ear discharge
253190015	Ear discharge symptom NOS
255620011	O/E - purulent ear discharge
4576851000006115	On examination - purulent ear discharge
5875381000006115	Finding of ear discharge
255623013	O/E - ear discharge NOS
253183013	Ear discharge symptoms
253186017	Ear discharge present
253225013	Has a sore throat
253227017	Sore throat symptom NOS
139761000006116	Sore throat
3206351000006111	Septic sore throat
398001015	Sore throat symptom
411477011	Persistent sore throat

286457014	Streptococcal sore throat NOS
286458016	Streptococcal sore throat with scarlatina NOS
1984341000006111	Manchester triage - Sore throat
61411000006110	Viral sore throat NOS
73157016	Streptococcal sore throat
121591000006115	Streptococcal sore throat with scarlatina
2164212016	Throat soreness
253248015	Throat symptom NOS
100191000006112	Throat symptom
253237010	Throat pain
253240010	Pain in throat
407058015	O/E - ear
255626017	O/E - ear auricle painful
12721121000006115	Streptococcal sore throat with scarlatina NOS
141055011	Streptococcal laryngitis
73158014	Streptococcal pharyngitis
69365016	Streptococcal tonsillitis
50618013	Scarlet fever
154821000006118	Scarlet fever - scarlatina
2724941000000115	Suspected scarlet fever
8466621000006112	Otitis media due to scarlet fever
5121451000006112	Staphylococcal scarlatina
879051000006113	Strep.throat + scarlatina NOS
50619017	Scarlatina
360178015	Fusobacterial necrotising tonsillitis
299517012	[X]Other infective otitis externa
298945018	Unspecified infective otitis externa
399494015	Acute infective otitis externa
6013861000006113	Infective otitis media
298974010	Infective otitis externa NOS
144257010	Infective otitis externa
298973016	Other specified infective otitis externa
298952016	Beach ear
298955019	Erysipelas - otitis externa
298954015	Infective otitis externa due to erysipelas
365556016	Infective otitis externa due to herpes simplex
4774451000006111	Infective otitis externa caused by impetigo
298959013	Infective otitis externa due to impetigo
822181000006114	Herpes simplex- otitis externa
822201000006110	Herpes zoster - otitis externa
298960015	Impetigo - otitis externa
298963018	Furunculosis of external auditory meatus
298966014	Chronic otitis externa due to aspergillosis

	298968010	Chronic mycotic otitis externa
	298969019	Other chronic infective otitis externa
	298970018	Chronic infective otitis externa
	56652016	Haemorrhagic otitis externa
	12732501000006112	Other otitis externa
	298975011	Other otitis externa
	299518019	[X]Other otitis externa
	658201000006118	External ear cholesteatoma
	131338019	Acute chemical otitis externa
	3380601000006111	Acute eczematoid otitis externa
	1788053014	Acute eczematoid otitis extern
	298983017	Other reactive otitis externa
	779301000006113	Inflammation ear external
	1231519019	Stenosis of external ear canal due to inflammation
	6204261000006117	Acute nonsuppurative otitis media
	458581000006110	Acute nonsuppurative otitis media NOS
	58707018	Acute otitis media with effusion
	7987351000006118	Acute bilateral otitis media with effusion
	473798017	Acute secretory otitis media
	4774551000006112	Acute serous otitis media
	3348911000006111	Acute mucoid otitis media
	128622017	Acute sanguinous otitis media
	3760591000006119	Acute sanguinous otitis media
	98475016	Acute allergic serous otitis media
	14738014	Acute allergic mucoid otitis media
	299022018	Bilateral chronic serous otitis
	299023011	Unilateral chronic serous otitis
	12450471000006110	Unilateral chronic serous otitis
	12027241000006113	Chronic serous otitis media of right ear
	135302019	Chronic serous otitis media
	299024017	Chronic serous otitis media
	12027231000006115	Chronic serous otitis media of left ear
	3826251000006118	Simple chronic serous otitis media
	130848015	Glue ear
	3782551000006114	Glue ear - mucoid
	3826261000006116	Glue ear - serous
	11759018	Mucosanguinous chronic otitis media
	3782531000006119	Simple chronic mucoid otitis media
	130846016	Chronic mucoid otitis media
	299032013	Chronic mucoid otitis media NOS
	299030017	Chronic mucoid otitis media
	5033691000006110	Chronic allergic otitis externa
	119572016	Chronic allergic otitis media

407501000006114	[X]Other chronic nonsuppurative otitis media
34701000006111	Other chronic nonsuppurative otitis media NOS
299039016	Allergic otitis media
299041015	Catarrhal otitis media
3884371000006117	Acute catarrhal otitis media
297581000006115	Nonsuppurative otitis media NOS
4774691000006119	ASOM - Acute suppurative otitis media
299061014	Acute suppurative otitis media
460541000006114	Acute suppurative otitis media - tympanic membrane intact
2736971000006114	Acute suppurative otitis media without spontaneous rupture of ear drum
4774711000006116	Acute suppurative otitis media due to another disease
3902951000006113	Acute suppurative otitis media with spontaneous rupture of ear drum
3902971000006115	Acute suppurative otitis media with discharge
299066016	Acute suppurative otitis media NOS
299065017	Acute suppurative otitis media due to disease EC
460551000006111	Acute suppurative otitis media - tympanic membrane ruptured
299068015	Purulent otitis media NOS
4774701000006119	Acute purulent otitis media
299069011	Bilateral suppurative otitis media
299071011	Acute left otitis media
299072016	Acute right otitis media
299073014	Acute bilateral otitis media
299534016	[X]Other mastoiditis and related conditions
5034051000006114	Acute mastoiditis and facial palsy
5034071000006116	Acute mastoiditis with intracranial complication
5034041000006112	Acute mastoiditis with facial paralysis
1480221019	Acute mastoiditis
299088016	Acute mastoiditis NOS
4197091000006115	Acute mastoiditis with complications
5034011000006113	Acute mastoiditis with neck abscess
469779010	Acute mastoiditis without complications
5034061000006111	Acute mastoiditis with labyrinthitis
299085018	Acute mastoiditis with complication
17974018	Abscess of mastoid
3672981000006114	Subperiosteal abscess of mastoid
2664101000006111	Abscess of mastoid
8694011	Empyema of mastoid
2563201000006113	Empyema of mastoid
1233461016	Subperiosteal mastoid abscess

63283016	Gradenigo's syndrome
3110901000006113	Gradenigo syndrome
299090015	Chronic mastoiditis NOS
133785015	Chronic mastoiditis
12731911000006111	Mastoiditis NOS
109102019	Otorrhoea
883841000006110	Otorrhoea - discharging ear
399506010	Unspecified otorrhoea
4548111000006114	Discharging ear
44084019	Otorrhagia
299229016	Otorrhoea NOS
299233011	Unspecified otalgia
21238010	Referred ear pain
299234017	Otalgia NOS
27089013	Otalgia
2696381000006111	Ear pain, referred
136470019	Acute nasopharyngitis
26785019	Acute sinusitis
61668014	Sinusitis
113403018	Acute maxillary sinusitis
150861013	Acute frontal sinusitis
5035651000006115	Acute frontal sinusitis with osteomyelitis of frontal bone
112674013	Acute ethmoidal sinusitis
129311019	Acute sphenoidal sinusitis
567061000000117	Acute rhinosinusitis
7875021000006116	Acute sinusitis caused by virus
7875011000006112	Acute sinusitis caused by fungus
301013015	Other acute sinusitis
301014014	Other acute sinusitis NOS
301015010	Acute sinusitis NOS
9421011	Acute pansinusitis
301021014	Acute gangrenous pharyngitis
301023012	Acute ulcerative pharyngitis
301029011	Allergic pharyngitis
301025017	Acute pneumococcal pharyngitis
301022019	Acute phlegmonous pharyngitis
301026016	Acute staphylococcal pharyngitis
149482010	Tonsillitis
301036012	Acute follicular tonsillitis
2947421000006113	Follicular tonsillitis
301035011	Acute erythematous tonsillitis
4577601000006117	On examination - follicular tonsillitis

301038013	Acute catarrhal tonsillitis
301041016	Acute bacterial tonsillitis
301037015	Acute ulcerative tonsillitis
2729261000006116	Ulcerative tonsillitis
301042011	Acute pneumococcal tonsillitis
301044012	Acute bacterial tonsillitis NOS
301043018	Acute staphylococcal tonsillitis
2682541000006114	Staphylococcal tonsillitis
7576111000006118	Bacterial tonsillitis
4200131000006119	Pneumococcal tonsillitis
4780231000006116	RAT - Recurrent acute tonsillitis
3517641017	Recurrent acute streptococcal tonsillitis
301047017	Recurrent acute tonsillitis
301086018	Tracheopharyngitis
12717051000006112	Tracheopharyngitis
66718013	Chronic sinusitis
301214019	Chronic sinusitis NOS
301211010	Other chronic sinusitis
1229598015	Chronic rhinosinusitis
59944016	Chronic maxillary sinusitis
146480018	Maxillary sinusitis
99894013	Chronic frontal sinusitis
130650014	Frontal sinusitis
121629017	Chronic ethmoidal sinusitis
301210011	Recurrent sinusitis
7080681000006111	Chronic recurrent sinusitis
147320013	Chronic pansinusitis
301212015	Pansinusitis
12724231000006113	Chronic tonsil and adenoid disease NOS
301238015	Chronic tonsil and adenoid disease NOS
411475015	Adenoid disease - chronic
411474016	Tonsil disease - chronic
150766011	Chronic tonsillitis
348209011	Chronic adenoiditis
396096012	Tonsil and/or adenoid hypertrophy
348208015	Enlargement of tonsil or adenoid
301227015	Hypertrophy of tonsils and adenoids NOS
3583461000006119	Hypertrophy of tonsils AND adenoids
885091000006111	Hypertrophy of tonsils/adenoid
3254151000006114	Hypertrophy of tonsils
789291000006119	Hypertrophy of adenoids alone
4197871000006111	Hypertrophy of adenoids
301228013	Adenoid vegetations

	301229017	Chronic adenotonsillitis
	301230010	Other chronic diseases of tonsils and adenoids
	301237013	Other chronic diseases of tonsils and adenoids NOS
	110445017	Cicatrix of tonsil
	92044014	Tonsillar tag
	301234018	Caseous tonsillitis
	301235017	Lingular tonsillitis
	453207016	Cyst of tonsil
	5886861000006116	Peritonsillar cyst
	453208014	Tonsillar cyst
	301239011	Chronic tonsil disease NOS
	229081000006112	Peritonsillar abscess
	885111000006119	Peritonsillar abscess -quinsey
	25522012	Quinsy
	301295019	Pharyngeal disease unspecified
	230901000006114	Pharynx or nasopharynx cellulitis
	140751018	Parapharyngeal abscess
	30574019	Retropharyngeal abscess
	301299013	Pharynx or nasopharynx cyst
	301300017	Pharynx or nasopharynx abscess
	348194019	Vallecular cyst
	48067018	Cellulitis of larynx
	301801012	[X]Other acute sinusitis
	301803010	[X]Acute tonsillitis due to other specified organisms
	301213013	Other chronic sinusitis NOS
	301825016	[X]Other chronic sinusitis
	252424016	Respiratory symptoms NOS
	317381016	[D]Respiratory symptom, unspecified
**Community acquired pneumonia**	427671000006111	Pneumococcal infectious disease
	128731000006119	Streptococ pneumon/cause/disease classified/oth chapters
	56816017	Streptococcal pneumonia
	4781141000006110	Group B streptococcal pneumonia
	739941000006111	Pneumococcal lobar pneumonia
	301409015	Lobar pneumonia
	1772681000006120	Lobar pneumonia
	5887841000006120	Lobar pneumonia left upper lobe
	546451000006112	Chest infection - pneumococcal pneumonia
	5053421000006110	Pneumococcal pneumonia
	1780488019	Atypical pneumonia screening test
	690181000000115	Mycoplasma pneumoniae present

3259121000006120	Mycoplasma pneumoniae pneumonia
3453851000006120	Mycoplasma pneumoniae
683591000006114	Mycoplasma pneumoniae [PPLO] cause/dis classifd/oth chaptr
219291000006113	Pleuropneumonia-like organism (PPLO) infection
219921000006115	Pneumonia due to pleuropneumonia like organisms
4781201000006120	Pneumonia due to pleuropneumonia-like organism
399241000006111	Infection due to Mycoplasma pneumoniae
301357010	Pneumonia and influenza
78272011	Mycoplasma pneumonia
125510013	Viral pneumonia
301362011	Pneumonia due to respiratory syncytial virus
301363018	Viral pneumonia NEC
301364012	Viral pneumonia NOS
301368010	Other bacterial pneumonia
301370018	Pneumonia due to other specified bacteria
301375011	Pneumonia due to bacteria NOS
301376012	Bacterial pneumonia
301377015	Pneumonia due to other specified organisms
301382010	Pneumonia due to specified organism NOS
301394018	Pneumonia with other infectious diseases EC
301403019	Pneumonia with other infectious diseases EC NOS
301404013	Pneumonia with infectious diseases EC NOS
301408011	Infective pneumonia
301413010	Influenza with pneumonia, influenza virus identified
301414016	Influenza with pneumonia NOS
301430012	Other specified pneumonia or influenza
301431011	Pneumonia or influenza NOS
301686019	Abscess of lung with pneumonia
301809014	[X]Other viral pneumonia
301810016	[X]Pneumonia due to other aerobic gram-negative bacteria
301811017	[X]Other bacterial pneumonia
301812012	[X]Pneumonia due to other specified infectious organisms
301813019	[X]Pneumonia in bacterial diseases classified elsewhere
301814013	[X]Pneumonia in viral diseases classified elsewhere
301817018	Pneumonia
301818011	[X]Other pneumonia, organism unspecified
350051017	Atypical pneumonia
396104016	Pneumonia with infectious diseases EC
396105015	Bronchopneumonia
451130014	Basal pneumonia

454280014	Septicaemia due to streptococcus pneumoniae
1222332017	Chest infection - other bacterial pneumonia
1222333010	Chest infection - pneumonia organism OS
1229740013	Influenza with bronchopneumonia
1231962012	Pneumonia with pertussis
1231963019	Pneumonia with whooping cough
1232627018	Pneumonia due to parainfluenza virus
1479355018	Community acquired pneumonia
2164029012	Mycoplasmal pneumonia
2900359016	Sepsis due to Streptococcus pneumoniae
2872480010	Pneumonia due to Human metapneumovirus
219801000006119	Pneumonia due to adenovirus
219821000006112	Pneumonia due to Eaton's agent
219841000006117	Haemophilus influenzae pneumonia
219851000006115	Pneumonia due to haemophilus influenzae
219971000006119	Staphylococcal pneumonia
219991000006118	Pneumonia due to streptococcus, group B
220071000006117	Pneumonia with cytomegalic inclusion disease
220241000006115	Pneumonia with typhoid fever
220251000006118	Chickenpox pneumonia
546421000006115	Chest infection - influenza with pneumonia
546481000006116	Chest infection - unspecified bronchopneumonia
546491000006118	Chest infection - viral pneumonia
778871000006116	Influenza with pneumonia
885191000006112	Lobar -pneumococcal -pneumonia
885221000006117	Whooping cough pneumonia
885231000006119	Bronchopneumonia
885241000006112	Pneumonia NOS
885251000006114	Influenza + pneumonia
885271000006116	Pneumonia/influenza NOS
932231000006112	Bilateral pneumonia
1760561000006120	Streptococcus pneumoniae
1772691000006120	Left upper zone pneumonia
1772721000006110	Bilateral basal pneumonia
1772711000006120	Basal pneumonia
1772701000006120	Lingular pneumonia
1772771000006110	Bronchopneumonia
1772761000006110	Right upper zone pneumonia
1772751000006120	Right middle zone pneumonia
1772741000006120	Right lower zone pneumonia
1772731000006110	Left lower zone pneumonia
1787121000006120	Community acquired pneumonia

	2609031000000110	Influenza with pneumonia due to seasonal influenza virus
	2622071000006120	Cytomegaloviral pneumonia
	3047811000006120	Pneumonia due to Streptococcus
	3163841000006110	Adenoviral pneumonia
	3164871000006120	Influenzal bronchopneumonia
	3334901000006110	Pneumonia due to Escherichia coli
	3437661000006110	Unresolved pneumonia
	3465961000006110	Pneumonia in pertussis
	3555881000006120	Parainfluenzal pneumonia
	3555901000006110	Parainfluenza pneumonia
	3556351000006110	Acute eosinophilic pneumonia
	3613101000006110	Organised pneumonia
	3669971000006110	Infective pneumonia acquired prenatally
	4353381000006110	Acute bronchopneumonia
	4781311000006110	Varicella pneumonia
	5053611000006120	Infectious mononucleosis pneumonia
	5887701000006110	Left basal pneumonia
	5887681000006110	Lower lobe pneumonia
	5887781000006120	RLL - Right lower lobe pneumonia
	5887761000006110	Right basal pneumonia
	5887721000006120	Left lower lobe pneumonia
	5887711000006110	LLL - Left lower lobe pneumonia
	5887871000006110	Right middle lobe pneumonia
	5887821000006110	Left upper lobe pneumonia
	5887791000006110	Right lower lobe pneumonia
	5887901000006110	RUL - Right upper lobe pneumonia
	5887921000006110	Right upper lobe pneumonia
	6566931000006120	Lobular pneumonia
	6566941000006110	Bilateral bronchopneumonia
	6750331000006110	Bilateral pneumonia
	7253251000006110	Pneumonia due to Staphylococcus aureus
	7569441000006110	Streptococcus acidominimus or Streptococcus pneumoniae or Streptococcus morbillorum
	7699541000006120	Pneumonia caused by Human coronavirus
	7758011000006110	Streptococcus pneumoniae or Streptococcus viridans group
	8031971000006110	Chronic pneumonia
	12116881000006100	Infection caused by Streptococcus pneumoniae
Otitis media	5033851000006115	Chronic non-suppurative otitis media without effusion
	11759018	Mucosanguinous chronic otitis media
	14738014	Acute allergic mucoid otitis media
	22934019	Postmeasles otitis media

30177017	Acute allergic sanguinous otitis media
58707018	Acute otitis media with effusion
63478014	Chronic purulent otitis media
87124011	Acute non-suppurative otitis media - mucoid
98475016	Acute allergic serous otitis media
119572016	Chronic allergic otitis media
128622017	Acute sanguinous otitis media
130846016	Chronic mucoid otitis media
135302019	Chronic serous otitis media
299014013	Acute non-suppurative otitis media - serous
299022018	Bilateral chronic serous otitis
299023011	Unilateral chronic serous otitis
299024017	Chronic serous otitis media
299030017	Chronic mucoid otitis media
299032013	Chronic mucoid otitis media NOS
299034014	Chronic otitis media with effusion, other
299039016	Allergic otitis media
299040019	Serous otitis media
299041015	Catarrhal otitis media
299042010	Mucoid otitis media
299056012	Chronic otitis media with effusion
299061014	Acute suppurative otitis media
299065017	Acute suppurative otitis media due to disease EC
299066016	Acute suppurative otitis media NOS
299067013	Chronic suppurative otitis media
299068015	Purulent otitis media NOS
299069011	Bilateral suppurative otitis media
299070012	Recurrent acute otitis media
299071011	Acute left otitis media
299072016	Acute right otitis media
299073014	Acute bilateral otitis media
299529015	[X]Other chronic suppurative otitis media
299530013	[X]Otitis media in bacterial diseases classified elsewhere
299532017	[X]Otitis media in other diseases classified elsewhere
399498017	Otitis media
473798017	Acute secretory otitis media
490746013	Chronic otitis media with effusion, purulent
490749018	Chronic secretory otitis media, purulent
503932010	Chronic secretory otitis media, mucoid
504727012	Chronic secretory otitis media, serous
34701000006111	Other chronic nonsuppurative otitis media NOS

77591000006116	Non-suppurative otitis media
116801000006110	Purulent otitis media
149921000006113	Serosanguineous chronic otitis media
297571000006118	Non-suppurative otitis media with eustachian tube disorders
297581000006115	Nonsuppurative otitis media NOS
406091000006110	[X]Other acute nonsuppurative otitis media
407501000006114	[X]Other chronic nonsuppurative otitis media
458531000006114	Acute non-suppurative otitis media
458581000006110	Acute nonsuppurative otitis media NOS
460541000006114	Acute suppurative otitis media - tympanic membrane intact
460551000006111	Acute suppurative otitis media - tympanic membrane ruptured
555761000006114	Chronic otitis media with effusion, mucoid
555791000006118	Otitis media with effusion - serous
556811000006111	Chronic suppurative otitis media - atticoantral
556821000006115	Chronic suppurative otitis media - tubotympanic
883681000006115	Acute nonsupp. otitis media
883711000006119	Chr. nonsupp. otitis media NOS
883741000006115	Chronic purulent otitis media
906681000006117	[RFC] Otitis media
1806411000006119	Acute otitis media
2548641000006112	AOM - Acute otitis media
2622401000006115	Chronic adhesive otitis media
2622421000006113	Adhesive otitis media
2712661000006118	Post measles otitis media
2736971000006114	Acute suppurative otitis media without spontaneous rupture of ear drum
2836821000006119	Chronic otitis media
3118451000006116	CSOM - Chronic suppurative otitis media
3118471000006114	Otitis media with effusion - purulent
3118481000006112	Chronic otitis media with perforation
3132041000006117	Suppurative otitis media
3176151000006118	Chronic atticoantral suppurative otitis media
3348911000006111	Acute mucoid otitis media
3562731000006111	OM - Otitis media
3760591000006119	Acute sanguinous otitis media
3760601000006110	Acute non-suppurative otitis media with haemotympanum
3760621000006117	Acute non-suppurative otitis media - sanguinous
3760631000006119	Acute non-suppurative otitis media - bloody
3782601000006111	Otitis media with effusion
3782621000006118	Secretory otitis media

	3782651000006110	Chronic secretory otitis media
	3826291000006112	Chronic non-suppurative otitis media with effusion - serous
	3902951000006113	Acute suppurative otitis media with spontaneous rupture of ear drum
	3902971000006115	Acute suppurative otitis media with discharge
	3924831000006114	Chronic tubotympanic suppurative otitis media
	4774551000006112	Acute serous otitis media
	4774691000006119	ASOM - Acute suppurative otitis media
	5033801000006119	Recurrent acute suppurative otitis media
	5033811000006116	Recurrent acute non-suppurative otitis media
	5033821000006112	Chronic non-suppurative otitis media
	5033831000006110	Otitis media with effusion - sanguinous
	6013861000006113	Infective otitis media
	6204261000006117	Acute nonsuppurative otitis media
	7987351000006118	Acute bilateral otitis media with effusion
	7987391000006112	Perforation of tympanic membrane due to otitis media
	8025131000006113	Acute persistent otitis media
**Tonsillitis**	301041016	Acute bacterial tonsillitis
	301042011	Acute pneumococcal tonsillitis
	301043018	Acute staphylococcal tonsillitis
	301044012	Acute bacterial tonsillitis NOS
	29982014	Acute tonsillitis
	69365016	Streptococcal tonsillitis
	149482010	Tonsillitis
	150766011	Chronic tonsillitis
	255676015	O/E - follicular tonsillitis
	255674017	O/E - tonsils - quinsy present
	255672018	On examination - tonsils moderately enlarged
	255673011	O/E - tonsils grossly enlarged
	301035011	Acute erythematous tonsillitis
	301036012	Acute follicular tonsillitis
	301037015	Acute ulcerative tonsillitis
	301038013	Acute catarrhal tonsillitis
	301040015	Acute gangrenous tonsillitis
	301046014	Acute viral tonsillitis
	301047017	Recurrent acute tonsillitis
	301049019	Acute tonsillitis NOS
	301803010	[X]Acute tonsillitis due to other specified organisms
	348206016	Vincent's tonsillitis
	360178015	Fusobacterial necrotising tonsillitis
	3517641017	Recurrent acute streptococcal tonsillitis

	100111000006119	Throat infection - tonsillitis
	229081000006112	Peritonsillar abscess
	853251000006117	Recurrent tonsillitis
	855671000006111	Acute tonsilitis/pharyngitis
	885021000006114	Mild Tonsillitis
	885111000006119	Peritonsillar abscess -quinsey
	2947421000006113	Follicular tonsillitis
	3329901000006119	Viral tonsillitis
	4075181000006112	Peritonsillar cellulitis
	4075191000006110	Acute peritonsillitis
	4780151000006115	Acute exudative tonsillitis
	4780231000006116	RAT - Recurrent acute tonsillitis
	5886851000006118	Tonsillar adenitis
	5655881000006116	Inflamed tonsils
	5894161000006115	Enlarged tonsil
	5898801000006117	Exudate on tonsils
	5912901000006119	Acute lingual tonsillitis
	7576111000006118	Bacterial tonsillitis
**Group A streptococcus**	50618013	Scarlet fever
	264410017	Notification of scarlet fever
	154821000006118	Scarlet fever - scarlatina
	272494100000115	Suspected scarlet fever
	3067351000006116	Scarlet fever streptococcus toxin
	50619017	Scarlatina
	286458016	Streptococcal sore throat with scarlatina NOS
	286791012	Parascarlatina
	121591000006115	Streptococcal sore throat with scarlatina
	879051000006113	Strep.throat + scarlatina NOS
	5121451000006112	Staphylococcal scarlatina
	1805781000006111	Invasive Group A beta-haemolytic streptococcal disease
	2725191000000112	Suspected invasive Group A beta-haemolytic streptococcal disease
	6730111000006117	Invasive Group A beta-hemolytic streptococcal disease
	7371101000006113	Sepsis due to Streptococcus pyogenes
	5911251000006118	Streptococcus pyogenes infection
	3804041000006110	Streptococcus pyogenes
	2900360014	Sepsis due to Streptococcus group A
	1779621000006112	Infection due to Streptococcus group A
	7371131000006117	Sepsis caused by Streptococcus group A
	2168151000000110	Group A streptococcus infection
	3804061000006114	Group A streptococcus

	6730121000006113	Group A streptococcal infection, invasive
	6844531000006113	Group A Strep
	6878921000006114	Small-colony-forming beta-hemolytic group A streptococci
**Urinary tract infection**	217691000000114	Micturition frequency
	1490597011	Frequency of micturition
	215531000006114	Polyuria
	217701000000114	Urinary frequency
	794711000006117	Frequency of micturition
	82701015	Dysuria
	252850010	Vaginal discharge symptom
	183161000006111	Recurrent urinary tract infections
	459357017	Suspected UTI
	259734010	Urine examination
	70381000006114	Urine tests
	411652019	MSU - general
	74321000006115	Urinalysis - general
	411680017	Leucocytes in urine
	185189016	Urine culture
	1220776015	Urine microscopy
	384760015	Mid-stream urine sample
	140294012	Nonspecific urethritis
	304056012	Other specified nephritis, nephrosis or nephrotic syndrome
	304058013	Nephritis, nephrosis and nephrotic syndrome NOS
	779911000006110	Infections of kidney
	177021000006119	Renal infections
	105227013	Chronic pyelonephritis
	304062019	Chronic pyelitis
	304063012	Chronic pyonephrosis
	585761000006118	Nonobstructive reflux-associated chronic pyelonephritis
	39884018	Calculous pyelonephritis
	304067013	Chronic pyelonephritis NOS
	61206018	Acute pyelonephritis
	54753017	Acute pyelitis
	304070012	Acute pyonephrosis
	304071011	Acute pyelonephritis NOS
	304083013	Pyelonephritis and pyonephrosis unspecified
	304084019	Pyelonephritis unspecified
	304085018	Pyelitis unspecified
	304086017	Pyonephrosis unspecified
	304090015	Unspecified pyelonephritis NOS

304091016	Infection of kidney NOS
65119018	Cystitis
113331011	Acute cystitis
304177012	Chronic interstitial cystitis
8285011	Subacute cystitis
304186019	Chronic cystitis unspecified
123644013	Trigonitis
107972012	Follicular cystitis
1230922019	Cystitis in actinomycosis
304205011	Recurrent cystitis
304206012	Other specified cystitis
22733019	Cystitis cystica
304208013	Other cystitis NOS
304209017	Cystitis NOS
74071000006117	Urethritis due to non venereal causes
229141000006119	Periurethritis
396725014	Urethral and periurethral abscess
111786017	Urethral abscess
111789012	Urethral gland abscess
12972011	Periurethral cellulitis
304276013	Candidal urethritis
304277016	Other urethritis
304278014	Urethritis unspecified
304279018	Urethral syndrome NOS
1231907015	Skene's glands adenitis
148643012	Urethral meatitis
304282011	Urethral meatal ulcer
304286014	Other urethritis NOS
74781000006117	Urinary tract infection, site not specified
183141000006112	Recurrent urinary tract infection
502401000006117	Bacteriuria, site not specified
354790018	Asymptomatic bacteriuria
195791000006119	Pyuria, site not specified
216611000006114	Post operative urinary tract infection
304319014	Recurrent urinary tract infection
183171000006116	Recurrent UTI
304321016	Chronic urinary tract infection
113884018	Urinary tract infection
1209801015	Urosepsis
229791000006112	Persistent proteinuria, unspecified
304323018	Urinary tract infection, site not specified NOS
305203014	[X]Other cystitis
305207010	[X]Other urethritis

317511010	[D]Dysuria
317514019	[D]Dysuria NOS
292171000006114	[D] Urgency of micturition
318021013	[X]Painful micturition, unspecified

Supplementary Table S2. Denominator populations in the month of April for each fiscal year

Age (years)	2016/17 N (%)	2017/18 N (%)	2018/19 N (%)	2019/20 N (%)	2020/21 N (%)	2021/22 N (%)	2022/23 N (%)
4-<5	190052 (20.8)	189746 (20.9)	185183 (20.5)	186224 (20.7)	188093 (21.2)	186410 (21.5)	183595 (21.5)
3-<4	187506 (20.6)	183087 (20.1)	183964 (20.4)	186496 (20.7)	184932 (20.8)	181456 (20.9)	177469 (20.7)
2-<3	180756 (19.8)	181661 (20.0)	183910 (20.3)	183364 (20.3)	179410 (20.2)	175148 (20.2)	171014 (20.0)
1-<2	179007 (19.6)	181188 (19.9)	180266 (19.9)	177598 (19.7)	172959 (19.5)	168522 (19.4)	161825 (18.9)
0-<1	174027 (19.1)	173444 (19.1)	170430 (18.9)	167788 (18.6)	161484 (18.2)	155782 (18.0)	161639 (18.9)
Total	911348	909126	903753	901470	886878	867318	855542

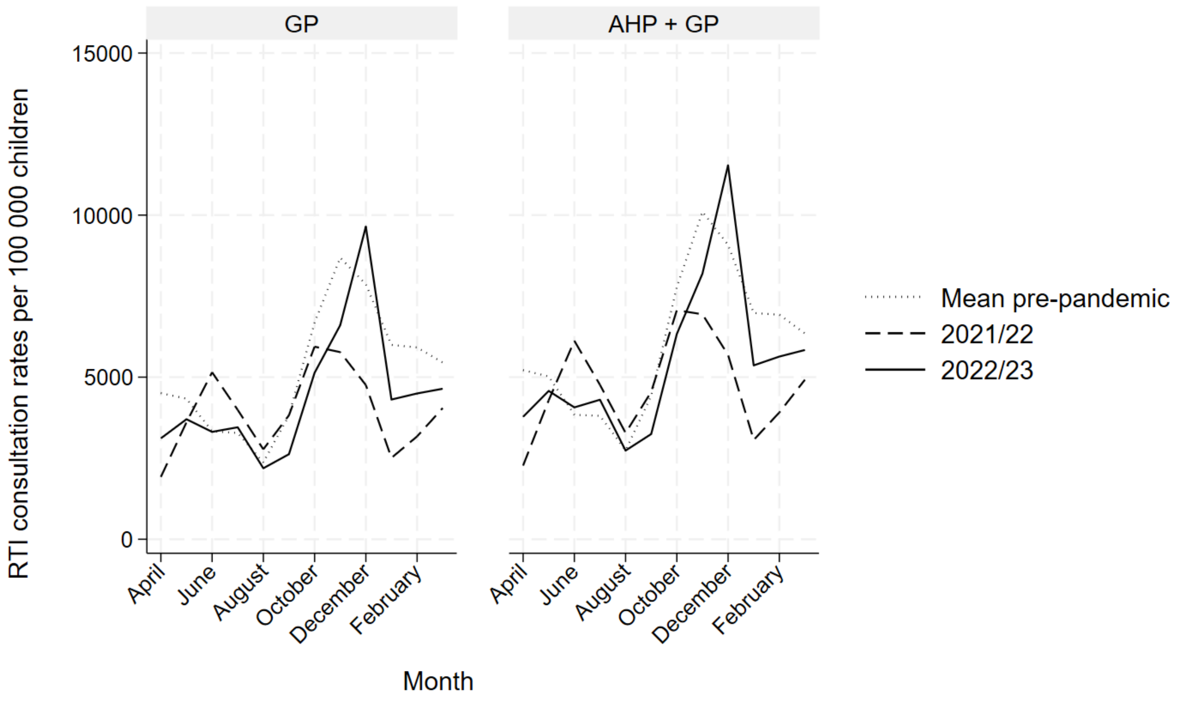

Figure S1. Trends in monthly consultation rates for RTIs in the pre-pandemic and post-pandemic lockdown periods (with and without the inclusion of AHPs by fiscal year, April to March)
